# Analysis of Genetic Code Ambiguity Arising from Nematode-Specific Misacylated tRNAs

**DOI:** 10.1371/journal.pone.0116981

**Published:** 2015-01-20

**Authors:** Kiyofumi Hamashima, Masaru Mori, Yoshiki Andachi, Masaru Tomita, Yuji Kohara, Akio Kanai

**Affiliations:** 1 Institute for Advanced Biosciences, Keio University, Tsuruoka, Japan; 2 Systems Biology Program, Graduate School of Media and Governance, Keio University, Fujisawa, Japan; 3 Genome Biology Laboratory, National Institute of Genetics, Mishima, Japan; 4 Department of Genetics, SOKENDAI, Mishima, Japan; 5 Faculty of Environment and Information Studies, Keio University, Fujisawa, Japan; Max-Planck-Institute for Terrestrial Microbiology, GERMANY

## Abstract

The faithful translation of the genetic code requires the highly accurate aminoacylation of transfer RNAs (tRNAs). However, it has been shown that nematode-specific V-arm-containing tRNAs (nev-tRNAs) are misacylated with leucine *in vitro* in a manner that transgresses the genetic code. nev-tRNA^Gly^ (CCC) and nev-tRNA^Ile^ (UAU), which are the major nev-tRNA isotypes, could theoretically decode the glycine (GGG) codon and isoleucine (AUA) codon as leucine, causing GGG and AUA codon ambiguity in nematode cells. To test this hypothesis, we investigated the functionality of nev-tRNAs and their impact on the proteome of *Caenorhabditis elegans*. Analysis of the nucleotide sequences in the 3’ end regions of the nev-tRNAs showed that they had matured correctly, with the addition of CCA, which is a crucial posttranscriptional modification required for tRNA aminoacylation. The nuclear export of nev-tRNAs was confirmed with an analysis of their subcellular localization. These results show that nev-tRNAs are processed to their mature forms like common tRNAs and are available for translation. However, a whole-cell proteome analysis found no detectable level of nev-tRNA-induced mistranslation in *C. elegans* cells, suggesting that the genetic code is not ambiguous, at least under normal growth conditions. Our findings indicate that the translational fidelity of the nematode genetic code is strictly maintained, contrary to our expectations, although deviant tRNAs with misacylation properties are highly conserved in the nematode genome.

## Introduction

The translation of genes into proteins is based on the genetic code, a set of essential rules for living cells. During protein translation, a complex is formed containing an amino acid and a transfer RNA (tRNA) in the activation reaction, which is catalyzed by aminoacyl-tRNA synthetase [1]. The resulting aminoacyl–tRNA is selected to participate in the translation process when its anticodon matches the codon on the messenger RNA (mRNA) in the ribosome, and the tRNA then transfers its amino acid to the growing polypeptide chain. The frequency of translational errors has been estimated to be approximately one misincorporated amino acid per 10,000 codons [2–5]. This faithful translation of the genetic code is a central pillar of molecular biology, and is maintained by the accuracy of tRNA aminoacylation and codon–anticodon matching. Therefore, any deviation from these sequential molecular recognition rules reflects the imperfection of the translation process, resulting in ‘genetic code ambiguity’ [6–8]. A number of instances of natural codon reassignment suggest that genetic code ambiguity has evolutionary implications. For instance, the UGA stop codon is ambiguous in several species within all three kingdoms of life, Bacteria, Archaea, and Eukarya, encoding the 21st proteinogenic amino acid, selenocysteine (Sec). The Sec residue is specifically incorporated into enzymes that are involved in oxidation–reduction reactions, by a recoding mechanism in a unique translational complex, the selenosome [9], and acts in their active sites. The UAG stop codon is also ambiguous in some methanogenic archaea and bacteria, encoding the 22nd proteinogenic amino acid, pyrrolysine, which like Sec, is inserted into the active centers of methyltransferases [10, 11]. Almost all genetic code ambiguity occurs at stop codons, and ambiguity at sense codons has only been found in several species of the genus *Candida*. The proteome of *Candida albicans* is unstable because deviant tRNAs carrying the CAG anticodon have a dual identity and decode the leucine (Leu) CUG codon not only as Leu but also as serine (Ser), with a frequency of ~5% [6, 7, 12]. Because these amino acid misincorporations alter the structural and biochemical characteristics of a large number of encoded proteins, the ambiguity of sense codons seems to have been strictly limited during the evolutionary diversification of most species.

Recent genome sequencing projects have identified a large number of eukaryotic tRNA isodecoders, tRNA molecules that share the same anticodons but differ in their molecular sequences, which sometimes contribute to the genome architecture and evolution [8, 13]. Several genomes of nematodes in the genus *Caenorhabditis* encode tRNA^Gly^ (CCC) and tRNA^Ile^ (UAU) molecules with an additional stem–loop structure in the variable region [14], called the “variable arm” (V-arm), although tRNAs containing the V-arm are generally restricted to tRNA^Leu^ and tRNA^Ser^ in eukaryotes [15]. In a previous study, we designated these “nematode-specific V-arm-containing tRNAs” (nev-tRNAs) and demonstrated their weak expression in *C. elegans* [8, 16]. *In vitro* aminoacylation assays have clearly shown that these tRNAs are solely charged with Leu, instead of glycine (Gly) or isoleucine (Ile). This is primarily attributable to the V-arm domains of the nev-tRNAs, which are very similar to that of tRNA^Leu^ and are known to be a major determinant of recognition by leucyl-aminoacyl tRNA synthetase [17–19].

In contrast, each nematode species also expresses tRNA^Gly^ (UCC) and tRNA^Ile^ (UAU) with standard short variable loops, which are the cognate tRNAs of nev-tRNA^Gly^ (CCC) and nev-tRNA^Ile^ (UAU), respectively. These cognate tRNAs decode the GGG codon and AUA codon, respectively, but tRNA^Gly^ (UCC) is charged with Gly by glycyl-tRNA synthetase and tRNA^Ile^ (UAU) with Ile by isoleucyl-tRNA synthetase [16]. Therefore, if nev-tRNA^Gly^ (CCC) participates in translation, GGG codons could be translated as either Gly or Leu in the competition between the nev-tRNA and its cognate tRNA. Similarly, if nev-tRNA^Ile^ (UAU) participates in translation, the AUA codon could be translated as either Ile or Leu. Importantly, cell-free protein expression assays have demonstrated that nev-tRNAs can be incorporated into eukaryotic ribosomes and used in protein synthesis and therefore cause genetic code ambiguity, at least *in vitro* [16]. This raises several fundamental questions. Typically, many tRNA genes are encoded as precursor forms in the genome, and they must be processed to yield mature functional forms after transcription. The processing steps include trimming, CCA addition, intron splicing, base modification, and nuclear export [20]. Although nev-tRNAs have unusual structural and aminoacyl properties that are inconsistent with the universal rules, it is unclear whether they are processed normally for translation like common tRNAs and whether they function in protein synthesis *in vivo*, which would confirm the ambiguity of the nematode genetic code.

To address these two questions, we first analyzed the functionality of nev-tRNAs in terms of their maturation by the addition of the 3′ CCA and their subcellular localization in *C. elegans*. Our results show that nev-tRNAs are weakly expressed but mature normally, and are exported from the nucleus like their cognate tRNAs. We next performed a large-scale analysis of amino acid misincorporation using high-resolution mass spectrometry (MS) to investigate whether nev-tRNAs participate in translation. However, no level of nev-tRNA-induced mistranslation was detected in the whole-cell proteome. This finding suggests that the nematode genetic code is not ambiguous, at least under normal growth conditions, and ensures high translational fidelity, contrary to our expectations.

## Results and Discussion

### Expression, maturation, and subcellular localization of nev-tRNAs

We have previously identified nematode-specific novel tRNA genes, designated nev-tRNAs, e.g., nev-tRNA^Gly^ (CCC) and nev-tRNA^Ile^ (UAU), which contain 15–20-nt V-arm structures and are solely charged with Leu instead of Gly or Ile *in vitro* [8, 16]. To obtain further evidence of the functionality of nev-tRNAs in cells, the following two characteristics were analyzed: (1) their maturation, with the addition of 3′ CCA; and (2) their subcellular localization. In these experiments, tRNA^Gly^ (UCC) and tRNA^Ile^ (UAU), which are the cognate tRNAs of nev-tRNA^Gly^ (CCC) and nev-tRNA^Ile^ (UAU), were used as the positive controls to test for GGG and AUA codon ambiguity in nematode cells.

**Figure 1:**
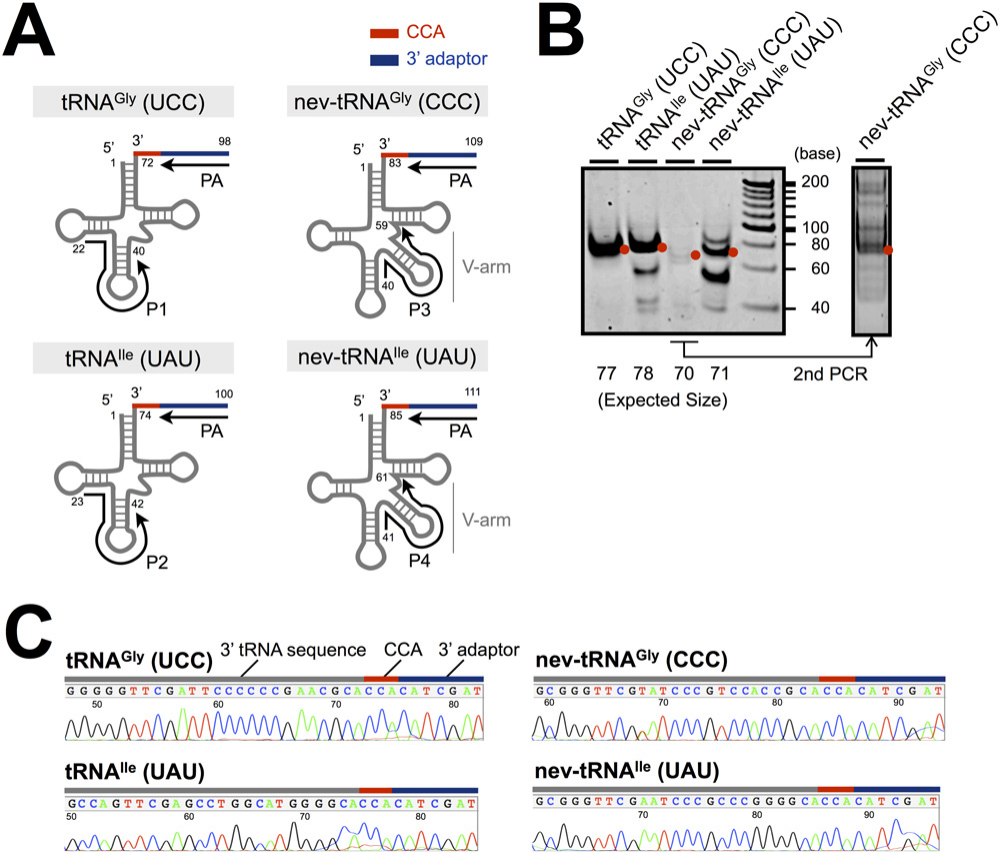
Detection of the 3′ CCA end sequences of nev-tRNAs. (A) PCR scheme for the detection of the 3′ ends of mature tRNAs: nev-tRNA^Gly^ (CCC) and nev-tRNA^Ile^ (UAU) and their cognates, tRNA^Gly^ (UCC) and tRNA^Ile^ (UAU), respectively. Numbers indicate the nucleotide positions relative to the 5′ end of each tRNA. (B) RT–PCR amplification of the 3′ end of each tRNA. PCR products of the expected sizes are shown as red dots. (C) Nucleotide sequence chromatograms of the 3′ end region of each tRNA.

The addition of CCA to the 3′ end of the tRNA molecule is one of its most important posttranscriptional modifications, and is essential for various tRNA functionalities, including other processing, aminoacylation, and tRNA–ribosome interactions [20]. To determine the 3′ end sequences of the nev-tRNAs with reverse transcription PCR (RT–PCR), a set of template tRNAs was isolated from mixed stages of *C. elegans* (eggs, larval stages 1–4, and adults), and ligated with a 23-nt adaptor sequence at their 3′ ends. RT–PCR amplification was conducted with forward primers that annealed to a specific region on each tRNA (positions 22–40 and 23–42 for common tRNA^Gly^ and tRNA^Ile^, respectively; positions 40–59 and 41–61 for nev-tRNA^Gly^ and nev-tRNA^Ile^, respectively) and reverse primers that annealed to the 3′ adaptor region (Fig. 1A). Targeted regions of the predicted lengths were successfully amplified, except for nev-tRNA^Gly^ (Fig. 1B). The amplification efficiency for nev-tRNA^Gly^ was considerably lower than that for the other templates but the amplified product was clearly detected with a second PCR analysis. The amplification efficiency for nev-tRNA^Ile^ was also slightly lower than those for the normal tRNAs. Taken together with our previous studies [16], these data suggest that the abundance of the mature nev-tRNAs in the cells was low. The amplified products of the expected sizes were then subcloned and the nucleotide sequences at their 3′ ends were determined. Fig. 1C shows that not only the common tRNA^Gly^ (UCC) and tRNA^Ile^ (UAU) but also nev-tRNA^Gly^ (CCC) and nev-tRNA^Ile^ (UAU) matured normally, with the addition of CCA at their 3′ ends. These findings show that nev-tRNAs are processed to the functional form for translation, just like their cognate tRNAs, although the structural and biochemical properties of the nev-tRNAs differ from those of normal tRNAs.

**Figure 2:**
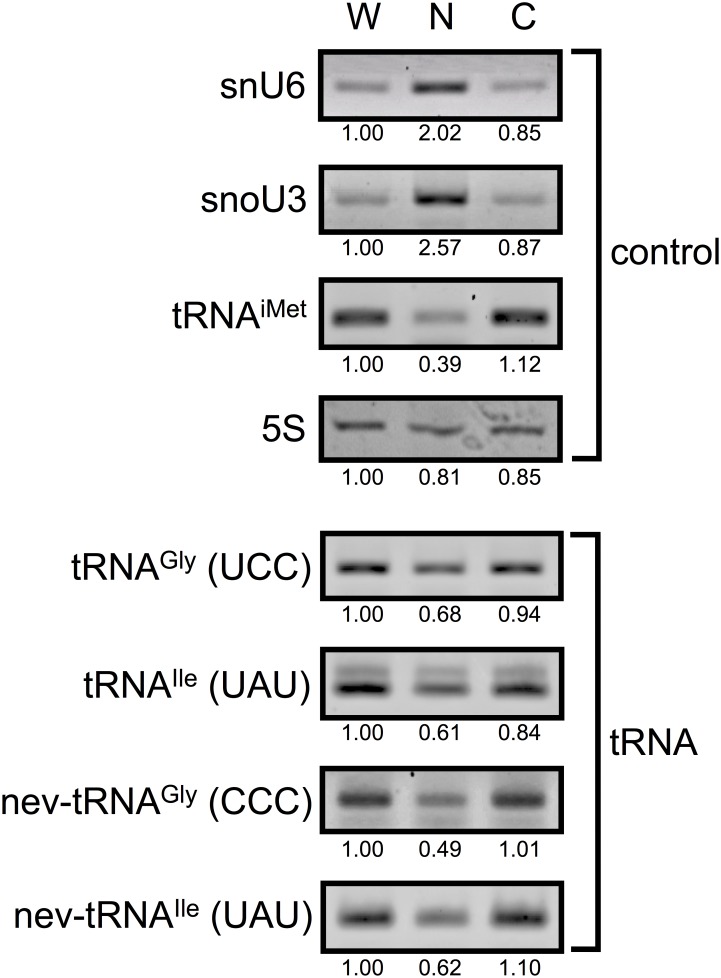
Subcellular localization of nev-tRNAs in *C. elegans*. RNA was isolated from each fraction of *C. elegans*: whole cell (W), nuclear (N), or cytoplasmic (C). RT–PCR analysis was used to detect snU6 and snoU3 RNAs (nuclear markers), tRNA^iMet^ (cytoplasmic marker), and four tRNAs (nev-tRNA^Gly^ and nev-tRNA^Ile^, and their cognate tRNAs). 5S rRNA expression is shown as the loading control. Band densities were evaluated semiquantitatively with densitometry.

We next analyzed the subcellular localization of the nev-tRNAs to determine whether they are exported from the nucleus after posttranscriptional modification. The whole *C. elegans* worm was subjected to subcellular fractionation with differential centrifugation (see Materials and Methods). Fig. 2 (upper panel) shows the subcellular localization of the control RNAs: U6 small nuclear RNA (snU6) and U3 small nucleolar RNA (snoU3) were enriched in the nucleus (~2.9-fold) relative to their levels in the cytoplasm, whereas tRNA^iMet^ was enriched in the cytoplasm (~2.8-fold) relative to its level in the nucleus, as previously reported [21, 22]. Under the same conditions, nev-tRNA^Gly^ (CCC) and nev-tRNA^Ile^ (UAU) were detected at higher levels (~2.0-fold) in the cytoplasm than in the nucleus (Fig. 2, lower panel), suggesting that the nev-tRNAs are exported from the nucleus and might therefore be used in translation. This experiment also confirmed that normal tRNA^Gly^ (UCC) and tRNA^Ile^ (UAU) are exported from the nucleus. Moreover, we determined the anticodon sequences of approximately 30 clones of each nev-tRNA, both in the nucleus and cytoplasm, and found that no anticodon was changed to a leucine codon by an RNA editing event. These results support the possibility that nev-tRNAs compete with their cognate tRNAs during translation. It must be noted that it is still unclear whether nev-tRNA anticodons are changed by specific chemical modifications so that they can read leucine codons.

### Analysis of amino acid misincorporation in the whole-cell proteome of *C. elegans*

Our previous studies have shown that nev-tRNA^Gly^ (CCC) can be incorporated into ribosomes and used for protein synthesis in an insect cell-free protein expression system [16]. This finding is evidence that nev-tRNAs cause genetic code ambiguity, at least *in vitro*. Because nev-tRNAs are exported from the nucleus and might compete with their cognate tRNAs in *C. elegans*, we assumed that nev-tRNAs are involved in protein synthesis *in vivo*, creating genetic code ambiguity. To address this hypothesis, we performed a shotgun proteomic analysis of *C. elegans* using liquid chromatography–tandem MS (LC–MS/MS), and examined the kinds of protein molecules within the whole-cell proteome that contained misincorporated amino acids. High-resolution MS can directly monitor very low levels of minor protein isoforms on a large scale [23, 24]. In this experiment, we mainly focused on Gly-to-Leu (in which Gly at the GGG codon is replaced with Leu) and Gly-to-Ser (in which Gly at the GGG codon is replaced with Ser) misincorporations. Gly-to-Ser misincorporations were used as the negative control because nev-tRNA^Gly^ (CCC) cannot be completely charged with Ser *in vitro* [16], suggesting that it does not cause Gly-to-Ser misincorporation. We did not look for Ile-to-Leu (in which Ile at the AUA codon is replaced with Leu) misincorporation because the Leu residue is indistinguishable from the Ile residue on MS, because they are structural isomers with identical molecular weights.

**Figure 3:**
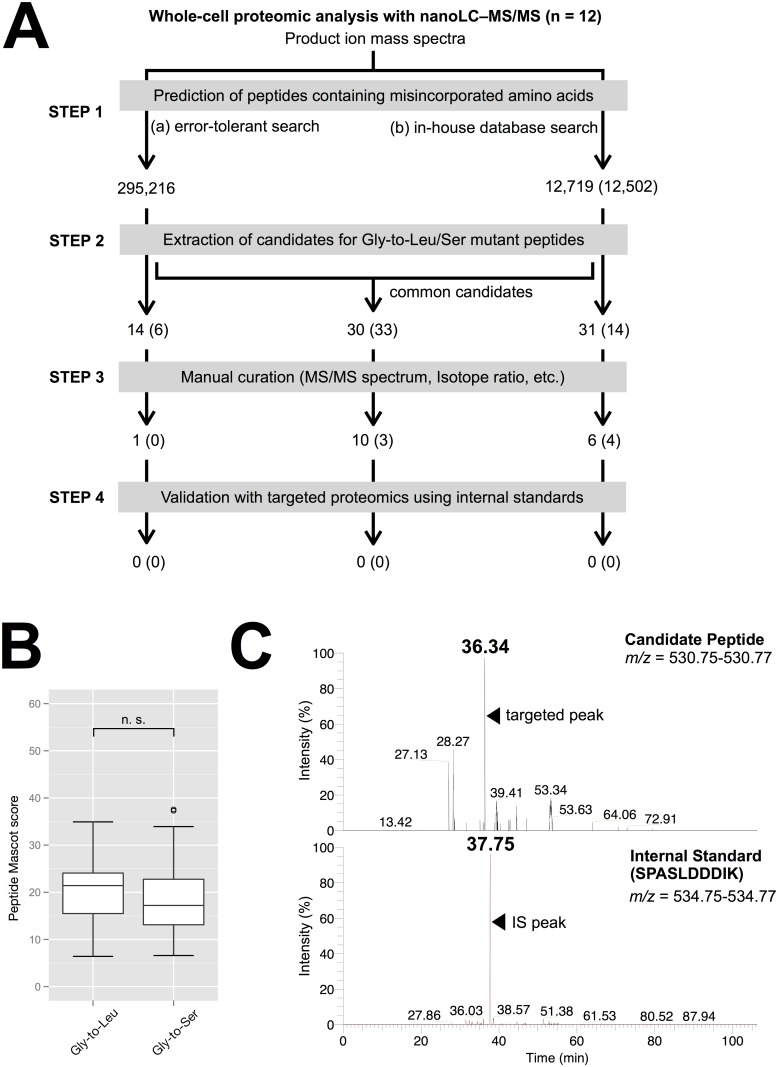
Screening for mutant peptides resulting from nev-tRNA^Gly^-dependent decoding. (A) Summary of the whole-cell proteome analysis of mixed-stage *C. elegans*. Values are the unique peptide counts at each step. Values in parentheses are the count of candidate peptides containing misincorporated Ser at the Gly (GGG) codon (negative control). (B) Boxplot of the confidence scores for the candidate peptides in Step 2. Significant differences were determined with Student’s two-sided *t* test. (C) Example of the validation of targeted proteomics. Extracted ion chromatograms of the candidate peptide and the synthetic peptide SPASLDDDIK (an internal standard) are shown. The candidate peptide ion was separated > 1.0 min earlier than the internal standard, indicating that the amino acid sequence of the candidate peptide was inconsistent with the sequence SPASLDDDIK.

For the whole-cell proteomic analysis, a protein mixture was extracted from mixed-stage *C. elegans* and fragmented into small peptides by digestion with site-specific enzymes. After the LC–MS/MS analysis of the resulting peptides, the data were examined with Mascot v2.4 (Matrix Science, London) to identify the amino acid misincorporations, using two different approaches: (a) an error-tolerant search; and (b) an in-house database search (Fig. 3A). The error-tolerant search is one of the optional modes of the Mascot protein database search [25], in which the raw data are initially searched against a reference protein database, after which the MS/MS data that do not match the expected amino acid sequences of known proteins are checked against a database containing all possible amino acid misincorporations and posttranslational modifications. With the error-tolerant search, 295,216 nonredundant (unique) peptides were identified. The in-house database search was developed and optimized in this study to compare the raw data against modified protein databases containing only possible Gly-to-Leu or Gly-to-Ser misincorporations, with no initial search against a reference protein database. This search identified 12,719 and 12,502 unique peptides, respectively (Fig. 3A, Step 1).

After discarding the low-quality peptides, 75 (= 14 + 30 + 31) candidate Gly-to-Leu mutant peptides and 53 (= 6 + 33 + 14) candidate Gly-to-Ser mutant peptides were extracted (Fig. 3A, Step 2). The mean Mascot confidence score for the Gly-to-Leu candidates was 20.3 ± 6.3, which did not differ significantly from that of the Gly-to-Ser candidates (*p* > 0.01) (Fig. 3B). The candidate misincorporations were then further screened by the manual curation of their MS/MS spectra and isotope ratios, and 17 (= 1 + 10 + 6) and seven (= 0 + 3 + 4) mutant peptides were finally obtained, respectively (Fig. 3A, Step 3, and summarized in S1 Table). To confirm that these peptides had identical amino acid sequences to those predicted with Mascot, a targeted proteome analysis was performed using an internal standard (IS) (Fig. 3A, Step 4). The IS was a synthesized peptide consisting of the same amino acid sequence as that identified with Mascot, in which one amino acid at the N- or C-terminus was labeled with a stable isotope (summarized in S2 Table). If the ions of both targeted peptides and the IS were detected at quite similar elution times with LC, indicating their almost equivalent chemical properties, peptide identification was deemed to be reliable. However, if their elution times differed by > 1.0 min, peptide identification was deemed to be unreliable. Validation with these criteria revealed that all the candidate misincorporations were false-positive Mascot identifications. One example is shown in Fig. 3C. This result means that no Gly-to-Leu mutant peptide was detectable, which was also true for the Gly-to-Ser negative control, suggesting that nev-tRNA^Gly^ (CCC) does not cause GGG codon ambiguity in the whole-cell proteome of *C. elegans*. This was also supported by the finding that no Gly-to-Leu candidate had a significantly higher Mascot score than the Gly-to-Ser candidates (Fig. 3B).

**Figure 4:**
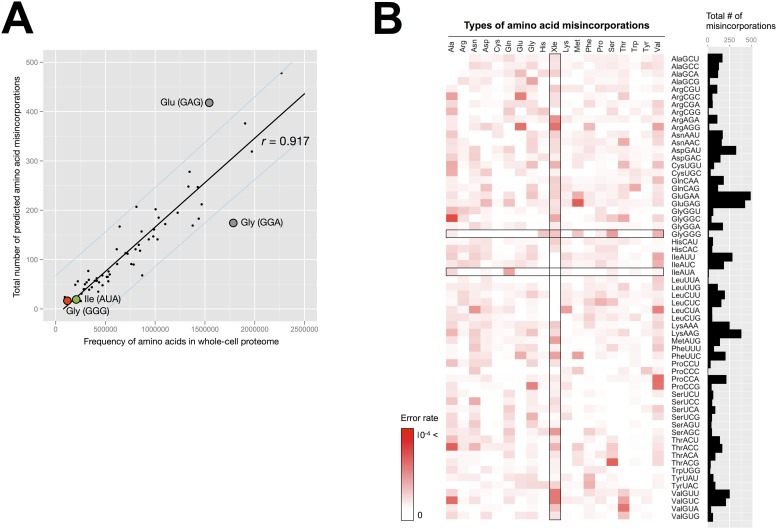
Distribution of amino acid misincorporations predicted in the whole-cell proteome of *C. elegans*. (A) Scatterplot of the frequencies of amino acids contained in all nonredundant peptides identified with a normal database search (x-axis) versus the total number of predicted amino acid misincorporations (y-axis) for each codon. The black line in the center denotes the linear regression line. The outer, light blue lines denote the 95% confidence interval for an individual predicted value. The red and green dots correspond to the GGG and AUA codons, respectively. The dots located outside the 95% confidence interval are shown in gray. (B) Heat map indicating the degree of predicted amino acid misincorporation (error rate) for each codon. The error rate was predicted by calculating the abundance of misincorporated amino acids relative to the total number of amino acids contained in the whole proteome. The matrix plots in the Gly (GGG) and Ile (AUA) row and in the ‘Xle’ (i.e., Ile or Leu) column are boxed. The total numbers of predicted misincorporations for each codon are indicated as a bar chart.

To gain more insight into the frequencies and variations of the amino acid misincorporations for each codon, we estimated the entire 64 × 19 possible codon-to-amino acid errors using data obtained with the error-tolerant search. Note that only a proportion of the identifications, with high Mascot confidence scores (> 30), was selected for this analysis because false-positive Gly-to-Leu misincorporations had low Mascot confidence scores (< 30), as described above. When the relationship between the amino acids used in the whole proteome and the number of predicted misincorporations for each codon was investigated with Pearson’s correlation coefficient, a strong significant correlation (*r* = 0.917) was observed (Fig. 4A). For example, the number of predicted misincorporations at frequent codons, such as the Glu (GAA) and Asp (GAU) codons, was up to 478, whereas fewer misincorporations were predicted at the Gly (GGG) and Ile (AUA) codons (approximately 4%). Furthermore, the Gly residues at the GGG codon showed little tendency to be substituted, not only with Leu (described as ‘Xle’ in the figure) but also with other amino acids (Fig. 4B). Similarly, there was no specific variation in the predicted misincorporations at the AUA codon. These observations show that nev-tRNAs do not seem to be involved in mistranslation at the corresponding codons in whole cells of *C. elegans*. However, in a single regression analysis, a dot corresponding to the Glu (GAG) codon was located outside the 95% confidence interval (Fig. 4A). As shown in Fig. 4B, Glu residues at the GAG codon tend to be substituted with Met residues at high levels (~7.3 × 10^–4^). In bacterial, yeast, and mammalian cells, it has been reported that Met is misacylated to specific nonmethionyl tRNA families, such as tRNA^Glu^ and tRNA^Lys^, and that these Met-misacylated tRNAs are used for protein synthesis during some cellular responses [26–29]. Although nev-tRNAs cannot decode the GAG codon because at least one base pair is mismatched, the common tRNA^Glu^ (CUC) encoded in the *C. elegans* genome can decode it. Therefore, the high Glu-to-Met error rate in *C. elegans* suggests the involvement of tRNA^Glu^ (CUC) misacylation in this phenomenon, as in bacterial, yeast, and mammalian cells.

### Possible explanations of the lack of genetic code ambiguity in *C. elegans*

We considered two possible reasons why no Gly-to-Leu mutant peptides were detected in this study, even though the nev-tRNAs matured normally and were exported from the nucleus. First, it is possible that nev-tRNAs are excluded from the protein synthesis process by a translation quality control mechanism. In bacteria, one of the elongation factors, EF-Tu, selectively binds to the correct aminoacyl-tRNAs and delivers them into the A-site of the ribosome [3, 30, 31]. In human neural cells, if the translation process is stopped because a tRNA is mutated, one of the ribosome release factor, GTPBP2, interacts with the ribosome recycling protein Pelota, and releases the stalled ribosome [32]. Although it is unclear whether homologues of EF-Tu and GTPBP2 act in *C. elegans*, as has been reported in other species, these findings allow the possibility that the translational errors induced by mischarged nev-tRNAs might be prohibited by such quality control systems.

Second, it is also possible that nev-tRNAs are used for protein synthesis in the cell, but that the frequency of amino acid misincorporations is below the level of MS detection. The MS-based method can directly measure a large number of amino acid misincorporations, down to a level of 0.01% (10^–4^) [23, 24]. However, because the abundance of mature nev-tRNAs in the cell is very low and they compete with highly expressed cognate tRNAs (Fig. 2), the incorporation of nev-tRNAs into ribosomes might be a rare and limited event compared with the incorporation of their cognate tRNAs. In addition to the low abundance of nev-tRNAs, we noted the low usage of the codons with which nev-tRNAs are associated. For instance, the GGG codon to which nev-tRNA^Gly^ (CCC) corresponds is the second rarest codon (0.44%) in *C. elegans* [16]. Therefore, we assume that even if nev-tRNAs participate in translation, the identification of amino acid misincorporations at the GGG codon is statistically more difficult than at other more frequent codons. This hypothesis is supported by the observation of more abundant misincorporations at the more frequent codons (Fig. 4A). Collectively, our data demonstrate that there is no mutant protein containing misincorporated Leu at “high” frequency in the whole-cell proteome, whereas it is still unknown whether such Leu residues are misincorporated into low-abundance proteins and/or some specific sites in proteins at low frequency.

To determine whether nev-tRNA-induced mistranslations can occur at low frequencies, an overexpressed single recombinant protein was analyzed with targeted proteomics. In this experiment, we overexpressed a green fluorescent protein (GFP)–LacZ protein and purify it to improve the detectable level of Gly-to-Leu misincorporation, because (i) the total 1284 codons of the *GFP–LacZ* mRNA contain 12 GGG codons (approximately 1% of the codons); and (ii) the purified samples for MS include a small number of proteins, mainly GFP–LacZ, resulting in low background noise. For this analysis, we constructed a transgenic strain expressing *myo-3p::GFP-LacZ* and extracted the protein mixture. After immunoprecipitation with an anti-GFP antibody, the purified GFP–LacZ protein was fragmented into small peptides by digestion with site-specific enzymes. The LC–MS/MS analysis was performed using two types of ISs for calibration, a synthetic peptide consisting of the same amino acid sequence as that in the database, and a synthetic peptide containing the Leu residue substituted for the Gly residue at the GGG codon (summarized in S3 Table). As shown in S4 Table, wild-type peptides containing the Gly residue at the GGG codon were detected at almost identical elution times as the ISs. In contrast, no aberrant peptide containing a misincorporated Leu residue at the GGG codon was detected. The fragmentation pattern in the mass spectrum of the identified peptide was consistent with that of the wild-type peptide rather than the aberrant peptide. One example is shown in S1 Fig. This result means that the Gly-to-Leu mutant peptides were not represented, even in the high-resolution targeted MS screen, suggesting that nev-tRNA^Gly^ (CCC) is not incorporated into ribosomes at a detectable level.

### Evolutionary implications of nev-tRNAs for the nematode genetic code

In this work, we have demonstrated that nev-tRNAs are weakly expressed, mature normally with the addition of the 3′ CCA, and are exported from the nucleus in *C. elegans*. However, no nev-tRNA-induced amino acid misincorporation was detected in the whole-cell proteome. The possible reasons include: (1) nev-tRNAs are not involved in translation; or (2) nev-tRNAs participate in translation but at a very low frequency. Consequently, the nematode genetic code does not seem to be ambiguous, although its genome contains these deviant tRNAs, which decode an alternative code. Because sense codon reassignment is strictly limited during evolution [6–8], nematode cells might actively regulate errors in protein synthesis with specific translational quality control mechanisms. Our observations provide an example of the robustness of the genetic code during translation, ensuring cellular homeostasis.

In contrast, pseudo-tRNA genes typically have several mismatched base pairings because of the high evolutionary rate [14, 33], but nev-tRNA genes do not contain such mutations and form a perfect cloverleaf secondary structure. The copy numbers of nev-tRNA genes and their anticodon variants have increased during the evolution of the nematode taxon [16]. From this feature of their evolutionary conservation, we also assume that they play important, if unexpected, roles, especially in certain biological processes. One such possible role is in the protective stress response. In bacterial, yeast, and mammalian cells, the level of Met-misacylation increases during the immune response, as described above. Because Met residues protect proteins from reactive oxygen species (ROS)-mediated damage [34], increased numbers of Met residues in proteins constitute a response mechanism, protecting cells against oxidative stress [29]. In addition to this pathway, recent studies have reported other putative benefits of mistranslation under stress conditions. In *Saccharomyces cerevisiae* cells, tRNA-misacylation-dependent translation errors increase the ubiquitylation and aggregation of proteins, and enhance the expression of heat shock proteins and other stress proteins. Consequently, the cells can survive even lethal environmental conditions [6, 7, 35]. Although nev-tRNAs are weakly expressed under normal growth conditions, their expression may be enhanced under some stress conditions, causing the synthesis of mistranslated proteins and the upregulation of the stress response to better cope with stress.

Another possible role of nev-tRNAs is in the gain of novel protein functions through the production of mutant proteins. Although most mistranslated proteins will probably be deleterious or neutral in function, a minority of these proteins will acquire novel or altered functions arising from their chemical and/or structural changes, including new subcellular localization [36], antibiotic resistance [37], or phenotypic diversification [38]. Although our data suggest that whole nematode cells do not synthesize mutant proteins using nev-tRNAs, it is still possible that some cells or tissues do synthesize such novel functional mistranslated proteins. For instance, there are cell-specific physiological differences in the translational error rate in mice [39]. Further studies are required to clarify the extensive expression patterns of nev-tRNAs under various environmental conditions and in different cells and tissues, and to identify the cellular response during the induction of genetic code ambiguity by nev-tRNAs.

## Materials and Methods

### Nematode culture and strain

The N2 strain of *Caenorhabditis elegans* and the OP50 strain of *Escherichia coli* used in this work were provided by the *Caenorhabditis* Genetics Center, which is funded by the NIH National Center for Research Resources. Mixed-stage worms, including eggs, larval stages 1–4, and adults, were grown with standard methods at 20°C [40].

A transgenic strain expressing *myo-3p::GFP-LacZ* was constructed by microinjecting wild-type worms with *myo-3p::GFP-LacZ* (pPD96.02) and coinjecting the marker *rol-6* (pRF4), generating an extrachromosomal array. We then randomly integrated the extrachromosomal array into the genome with UV irradiation [41]. The transgenic worms containing the integrated array (msIs4) were back-crossed twice to the wild type, generating strain YK38.

### Detection of the CCA sequence at the 3′ ends of tRNAs

Total RNA was isolated from mixed-stage *C. elegans* with TRIzol Reagent (Invitrogen, Carlsbad, CA, USA), according to the manufacturer’s instructions. The RNAs were separated on a denaturing 6% polyacrylamide gel containing 8 M urea, and the tRNA fraction, ranging from 60 to 90 nt, was purified from the gel. These RNAs were treated with bacterial alkaline phosphatase and ligated with an adaptor sequence CATCGATCCTGCAGGCTAGAGAC at their 3′ ends using the Small RNA Cloning Kit (TaKaRa, Shiga, Japan). The resulting RNAs were used as the templates for RT–PCR analysis.

To determine the sequences at the 3′ ends of the tRNAs, RT–PCR was performed with ReverTra Dash reverse transcriptase and KOD FX DNA polymerase (Toyobo Biochemicals, Osaka, Japan). The amplification reactions consisted of 30 cycles of denaturation at 98°C for 30 s, annealing at 60°C for 30 s, and extension at 74°C for 30 s, with specific primers (S5 Table; see also Fig. 1A). The PCR products were separated on 10% polyacrylamide gel and then purified from the gel to remove the primers, primer dimers, and nonspecific PCR products. The purified PCR products were subcloned into the pCR-Blunt II-TOPO vector (Invitrogen). The nucleotide sequences of the inserted DNAs were determined with an ABI3100 DNA Sequencer (Applied Biosystems, Foster City, CA, USA).

### Analysis of the subcellular localization of tRNAs

Whole *C. elegans* worms were subjected to subcellular fractionation according to the protocol of Zisoulis *et al*. [22], with slight modifications. Briefly, mixed-stage worms were harvested, snap frozen, and lysed in ice-cold NP-40 lysis buffer (NLB) containing 10 mM Tris-HCl (pH 7.4), 10 mM NaCl, 3 mM MgCl_2_, 0.5% NP-40, 1 mM dithiothreitol (DTT), and 100 U/ml RNasin Plus (Promega, Madison, WI, USA) using a mortar and pestle precooled with liquid N_2_. The lysates were centrifuged at 500 × g for 30 s at 4°C and one-fifth of the supernatant volume was collected (whole-cell fraction). The remainder was centrifuged at 2,000 × g for 5 min at 4°C. The supernatant (postnuclear fraction, i.e., cytoplasmic fraction) was centrifuged again (2,000 × g for 5 min at 4°C) and the pellet (nuclear fraction) was washed with NLB. The nuclear fraction was resuspended in a volume of NLB equivalent to that of the cytoplasmic fraction. The RNAs in each fraction were isolated with TRIzol Reagent and used as the templates for RT–PCR.

To examine the expression of tRNAs in each fraction, RT–PCR was performed with the enzymes ReverTra Dash and KOD FX Neo (Toyobo Biochemicals). The amplification reactions consisted of 20–35 cycles of denaturation at 98°C for 10 s, annealing at 55/60°C for 3 s, and extension at 74°C for 6 s, with specific primers (S6 Table). The PCR conditions were optimized by manipulating the number of cycles and the annealing temperature to determine the linear ranges. The PCR products were separated by electrophoresis on 3% NuSieve 3:1 Agarose gel (Cambrex Bio Science, Rockland, ME, USA). The bands were stained with ethidium bromide and visualized with a Molecular Imager FX Pro (Bio-Rad Laboratories, Hercules, CA, USA), and then semiquantified with densitometry using the ImageJ v1.48 software [42]. The RT–PCR products were then purified with the Illustra GFX PCR DNA and Gel Band Purification Kit (GE Healthcare, Buckinghamshire, UK), and subcloned into the pCR-Blunt II-TOPO vector (Invitrogen). The nucleotide sequences of the inserted DNAs were determined with an ABI3100 DNA Sequencer (Applied Biosystems), and confirmed to be identical to those in the database.

### Immunoprecipitation of the GFP–LacZ protein

The GFP–LacZ protein was immunoprecipitated with GFP-Trap_A (ChromoTek, Martinsried, Planegg, Germany) from mixed-stage transgenic worms expressing *myo-3p::GFP-LacZ*, according to the manufacturer’s instructions. The purified protein was detected with an immunoblotting analysis using an anti-GFP antibody (Clontech Laboratories, Mountain View, CA, USA, catalogue # 632380), and its molecular weight was identical to that in the database.

### NanoLC–MS/MS analysis

The protein mixture was extracted from mixed-stage wild-type worms with 100 mM triethylammonium bicarbonate (TEAB; pH 8.5) containing 12 mM sodium deoxycholate and 12 mM sodium dodecanoyl sarcosinate. The resulting mixture or the purified GFP–LacZ protein was reduced with 10 mM DTT at room temperature for 30 min, and alkylated with 47 mM iodoacetamide at room temperature in the dark for 30 min. The samples were diluted five-fold with 100 mM TEAB (pH 8.5) and digested with sequence-grade lysyl endoprotease (Lys-C), trypsin, endprotease Glu-C (V8), or chymotrypsin, and then desalted with StageTips using a C18 Empore disk membrane [43].

An LTQ-Orbitrap mass spectrometer (Thermo Fisher Scientific, Bremen, Germany) equipped with a nanoLC interface (Nikkyo Technos, Tokyo, Japan), a Dionex UltiMate 3000 pump with an FLM-3000 Flow Manager (Germering, Germany), and an HTC-PAL Autosampler (CTC Analytics, Zwingen, Switzerland) were used for the nanoLC–MS/MS measurements. Reprosil C18 material (3 μm; Dr Maisch, Ammerbuch, Germany) was packed into a self-pulled needle (100 μm, I.D. × 130 mm; tip I.D. 5 μm) with a nitrogen-pressurized column load cell (Nikkyo Technos, Tokyo, Japan) to prepare an analytical column needle with a “stone-arch” frit [44]. The flow rate was 500 nL/min. The mobile phases consisted of (A) 0.5% acetic acid and (B) 0.5% acetic acid and 80% acetonitrile. A three-step linear gradient was used: 5% to 10% B in 5 min, 10% to 40% B in 60 min, 40% to 100% B in 5 min, and maintained at 100% B for 10 min. A spray voltage of 2400 V was applied. The MS scan range was *m/z* 300–1500 in the Orbitrap mass spectrometer and the top 10 precursor ions (for whole-cell proteomics) or the targeted precursor ions (for targeted proteomics) were selected for subsequent MS/MS scans in the linear ion trap mass spectrometer.

### Identification of peptides containing misincorporated amino acids

Peak lists were created using an in-house Perl script based on the recorded product ion mass spectra. Peptides and proteins were identified with Mascot v2.4 (Matrix Science, London) against the UniProt/Swiss-Prot database (downloaded 2013/06, subset *C. elegans*, 3428 protein entries) in error-tolerant mode [25] or in an in-house protein database (in which all Gly residues arising from the decoding of the GGG codons were altered to Leu or Ser). A precursor mass tolerance of 3 ppm and a fragment ion mass tolerance of 0.8 Da were used with strict enzyme specificity, allowing for up to two missed cleavages [45]. The carbamidomethylation of cysteine was set as a fixed modification, and methionine oxidations were allowed as variable modifications. Peptides were rejected if the Mascot score was below the 95% confidence limit based on the “identity” score for each peptide. Some of the identified peptides were further validated with a targeted proteomics approach using synthesized peptides (AQUA Peptides; Sigma-Aldrich, St. Louis, MO, USA) that contained stable-isotope-labeled amino acids (see S2 and S3 Tables).

## Supporting Information

S1 Table Candidate peptides containing misincorporated Leu/Ser at Gly (GGG) codon. ^a^ Amino acid residues arising from the decording of Gly (GGG) codon are shown in red. ^b^ Screening types of each mutant peptide candidate are shown (see Fig. 3A). ^c^ Chromatographic retention times of the targeted peptides are shown.
(PDF)

S2 Table List of internal standards for the calibration of whole-cell proteomics. ^a^ Amino acid residues at the Gly (GGG) codon are shown in red. Stable isotopically labeled amino acids are underlined.
(PDF)

S3 Table List of internal standards used in targeted proteomic analysis of purified GFP–LacZ. ^a^ Amino acid residues at the Gly (GGG) codon are shown in red. Stable isotopically labeled amino acids are underlined.
(PDF)

S4 Table Summary of the identified peptides from transgenic worms expressing GFP–LacZ. The peptide sequences containing amino acid residues (red) arising from the decoding of the GGG codon are listed. ^a^ Chromatographic retention times of the targeted peptides are also shown.
(PDF)

S5 Table Oligonucleotides used to detect the CCA sequence at the 3′ end of each tRNA. (PDF)

S6 Table Oligonucleotides used to analyze the subcellular localization of tRNAs. ^a^ PCR reactions were performed using the indicated cycle numbers: denaturation for 10 s at 98°C, annealing for 3 s at the indicated temperatures and extension for 6 s at 74°C (See Materials and Methods for details).
(PDF)

S1 Fig Fragmentation pattern in the mass spectrum of the identified peptide from *C. elegans* is inconsistent with those of the peptides containing misincorporated Leu. MS/MS spectrum of the identified peptide from the GFP-LacZ proteins expressed in *C. elegans* was compared with those of synthesized peptides (IS) with the sequence SA(G/L)QLWLTVR. The C-terminal arginine residue of each IS was labeled with a stable isotope (Δm/z = 10.008269).
(PDF)
