# Supplementary material for: Analysis of Genetic Code Ambiguity Arising from Nematode-Specific Misacylated tRNAs
Source: PLoS One. 2015 Jan 20;10(1):e0116981. doi: 10.1371/journal.pone.0116981 (PMC4300185; doi:10.1371/journal.pone.0116981)
Supplement: S1 Table — a Amino acid residues arising from the decording of Gly (GGG) codon are shown in red. b Screening types of each mutant peptide candidate are shown (see Fig. 3A). c Chromatographic retention times of the targeted peptides are shown. (PDF) [file pone.0116981.s001.pdf]

Table S1. Candidate peptides containing misincorporated Leu/Ser at Gly (GGG) codon

Type	Sequence [a]	Modification	Observed *m/z*	Mascot Score	Screening [b]	Retention time (min) [c]			Source protein for identified peptide	
						Candidate	Internal Standard	Δ time	Swiss-Prot AC #	Description
Gly-to-Leu	LNLLLEVK		471.3051	23.67	common	70.1	59.5	10.6	MADD_CAEEL	MAP kinase-activating death domain protein
	QPGEEPPAGRGKGLKK		550.3103	14.13	common	60.8	25.2	35.6	MED1_CAEEL	Mediator of RNA polymerase II transcription subunit 1.1
	TALAVGTRR		472.7859	23.00	common	38.3	27.6	10.7	NDUS7_CAEEL	Probable NADH dehydrogenase [ubiquinone] iron-sulfur protein 7 mitochondrial
	ERGELLESGPK		607.8239	12.72	common	46.7	34.6	12.1	NGLY1_CAEEL	Peptide-N(4)-(N-acetyl-beta-glucosaminyl)asparagine amidase
	QVLCKDL		438.2366	17.49	common	ND	ND	-	PAFA_CAEEL	Platelet-activating factor acetylhydrolase homolog 2
	FEEAQRLR		524.7807	21.22	common	36.7	30.6	6.1	IFA2_CAEEL	Intermediate filament protein ifa-2
	AFYLAVMR		485.7629	19.27	common	57.1	58.3	1.2	LE418_CAEEL	Protein let-418
	RTDTLK		367.2139	12.70	common	ND	ND	-	RIM_CAEEL	Rab-3-interacting molecule unc-10
	ALEMISLK		452.7621	18.27	common	48.5	52.5	4.0	ACN1_CAEEL	Inactive angiotensin-converting enzyme-related protein
	RDLHINMAYL		623.3240	10.55	common	ND	54.9	-	CADH3_CAEEL	Cadherin-3
	LAELNNTLIISIL		713.9298	18.62	(a)	ND	ND	-	CLAP1_CAEEL	Protein CLASP-1
	EDAATSVKVANLL		665.8645	31.15	(b)	72.8	57.4	15.4	CHITL_CAEEL	Chitinase-like protein C25A8.4
	RSKDVLL		415.7591	23.69	(b)	40.8	31.2	9.6	SIR41_CAEEL	NAD-dependent protein deacetylase sir-2.2
	TPSMKVIL		444.7649	25.66	(b)	68.9	53.5	15.4	NHR20_CAEEL	Nuclear hormone receptor family member nhr-20
	SPASLDDDIK		530.7622	20.47	(b)	36.3	37.8	1.5	PS11A_CAEEL	Probable 26S proteasome regulatory subunit rpn-6.1
	LLIEK		308.2075	31.40	(b)	ND	ND	-	SRRT_CAEEL	Serrate RNA effector molecule homolog
	TLQIK		301.6973	25.82	(b)	ND	ND	-	CLH_CAEEL	Probable clathrin heavy chain 1
Gly-to-Ser	LSEEAILSF		336.8471	15.16	common	35.2	59.9	24.7	DHTK1_CAEEL	Probable 2-oxoglutarate dehydrogenase E1 component DHKTD1 homolog mitochondrial
	SGIIQATELSR		587.8251	27.20	common	ND	45.1	-	U520_CAEEL	Putative U5 small nuclear ribonucleoprotein 200 kDa helicase
	AEKQVDSLR		523.2858	22.76	common	34.1	27.0	7.1	HUTU_CAEEL	Probable urocanate hydratase
	HSLIR		313.1930	24.63	(b)	ND	ND	-	GALT9_CAEEL	Probable N-acetylgalactosaminyltransferase 9
	MPSLFPMK	Oxidation@M:7	483.7424	23.90	(b)	50.6	58.5	7.9	DGK3_CAEEL	Probable diacylglycerol kinase 3
	VTSQEGAR		424.2170	27.19	(b)	ND	22.6	-	SRB5_CAEEL	Serpentine receptor class beta-5
	FISSLPIIR		523.3242	26.38	(b)	73.9	58.8	15.1	SRE37_CAEEL	Serpentine receptor class epsilon-37

[a] Amino acid residues arising from the decording of Gly (GGG) codon are shown in red.

[b] Screening types of each mutant peptide candidate are shown (see Figure 3A).

[c] Chromatographic retention times of the targeted peptides are shown.
