# Supplementary material for: Analysis of Genetic Code Ambiguity Arising from Nematode-Specific Misacylated tRNAs
Source: PLoS One. 2015 Jan 20;10(1):e0116981. doi: 10.1371/journal.pone.0116981 (PMC4300185; doi:10.1371/journal.pone.0116981)
Supplement: S2 Table — a Amino acid residues at the Gly (GGG) codon are shown in red. Stable isotopically labeled amino acids are underlined. (PDF) [file pone.0116981.s002.pdf]

Table S2. List of internal standards for the calibration of whole-cell proteomics

Type	Name	Sequence [a]	Label	Calculated *m/z*
Gly-to-Leu	MADD_CAEEL_N	LNLLLEVK	L_C13N15	474.8140
	MADD_CAEEL_C	LNLLLEVK	K_C13N15	475.3125
	MED1_CAEEL	QPGEEPPAGRGKGLKK	K_C13N15	552.9809
	NDUS7_CAEEL	TALAVGTRR	R_C13N15	477.7898
	NGLY1_CAEEL	ERGELLESGPK	K_C13N15	611.8298
	PAFA_CAEEL	QVLCKDL	L_C13N15	413.2345
	IFA2_CAEEL	FEEAQRLR	R_C13N15	529.7847
	LE418_CAEEL	AFYLAVMR	R_C13N15	490.7670
	RIM_CAEEL	RTDTLK	K_C13N15	371.2211
	ACN1_CAEEL	ALEMISLK	K_C13N15	456.7696
	CADH3_CAEEL	RDLHINMAYL	L_C13N15	626.8329
	CLAP1_CAEEL	LAELNNTLIISIL	L_C13N15	717.4383
	CHITL_CAEEL	EDAATSVKVANLL	L_C13N15	669.3731
	SIR41_CAEEL_N	RSKDVLL	R_C13N15	419.2672
	SIR41_CAEEL_C	RSKDVLL	L_C13N15	420.7628
	NHR20_CAEEL	TPSMKVIL	L_C13N15	448.2736
	PS11A_CAEEL	SPASLDDDIK	K_C13N15	534.7688
	SRRT_CAEEL	LLIEK	K_C13N15	312.2148
	CLH_CAEEL	TLQIK	K_C13N15	305.7046
Gly-to-Ser	DHTK1_CAEEL	LSEEAILSF	L_C13N15	339.1859
	U520_CAEEL	SGIIQATELSR	R_C13N15	592.8294
	HUTU_CAEEL	AEKQVDSLR	R_C13N15	528.2899
	GALT9_CAEEL	HSLIR	R_C13N15	318.1970
	DGK3_CAEEL	MPSLFPMK	K_C13N15	479.7529
	SRB5_CAEEL	VTSQEGAR	V_C13N15	427.2242
	SRE37_CAEEL	FISSLPIIR	R_C13N15	528.3283

[a] Amino acid residues at the Gly (GGG) codon are shown in red. Stable isotopically labeled amino acids are underlined.
