# Supplementary material for: Analysis of Genetic Code Ambiguity Arising from Nematode-Specific Misacylated tRNAs
Source: PLoS One. 2015 Jan 20;10(1):e0116981. doi: 10.1371/journal.pone.0116981 (PMC4300185; doi:10.1371/journal.pone.0116981)
Supplement: S3 Table — a Amino acid residues at the Gly (GGG) codon are shown in red. Stable isotopically labeled amino acids are underlined. (PDF) [file pone.0116981.s003.pdf]

Table S3. List of internal standards used in targeted proteomic analysis of purified GFP–LacZ

Type	Name	Sequence [a]	Label	Calculated *m/z*
GGG-to-Gly	GFP2-Trp-G	DDGNYK	K_C13N15	711.2944
	GFP3-Trp-G	DHMVLLEFVTAAGITHGMDELYK	K_C13N15	2590.2680
	LacZ3-Trp-G	LSGQTIEVTSEYLFR	R_C13N15	1742.8959
	LacZ7-Trp-G	VNWLGLGPQENYPDR	R_C13N15	1757.8606
	GFP1-V8-G	VNGHKFSVSGE	V_C13N15	1160.5695
	GFP2-V8-G	GNYKTRAE	R_C13N15	938.4690
	GFP3-V8-G	FVTAAGITHGMD	V_C13N15	1219.5776
	LacZ2-V8-G	RNHPSVIIWSLGNE	R_C13N15	1621.8445
	LacZ7-V8-G	RVNWLGLGPQE	R_C13N15	1268.6746
	LacZ8-V8-G	NGLRCGTRE	R_C13N15	1062.5109
	LacZ9-V8-G	GFHMGIGGD	F_C13N15	890.3825
	LacZ1-TrpV8-G	GVNSAFHLWCNGR	R_C13N15	1517.7066
	LacZ4-TrpV8-G	SAGQLWLTVR	R_C13N15	1130.6317
	LacZ5-TrpV8-G	AGHISAWQQWR	R_C13N15	1339.6654
	LacZ6-TrpV8-G	AVLITTAHAWQHQGK	K_C13N15	1660.8918
GGG-to-Leu	GFP2-Trp-L	DDLNYK	K_C13N15	767.3570
	GFP3-Trp-L	DHMVLLEFVTAALITHGMDELYK	K_C13N15	2646.3306
	LacZ3-Trp-L	LSLQTIEVTSEYLFR	R_C13N15	1798.9585
	LacZ7-Trp-L	VNWLGLLPQENYPDR	R_C13N15	1813.9232
	GFP1-V8-L	VNLHKFSVSGE	V_C13N15	1216.6321
	GFP2-V8-L	LNYKTRAE	R_C13N15	994.5316
	GFP3-V8-L	FVTAALITHGMD	V_C13N15	1275.6402
	LacZ2-V8-L	RNHPSVIIWSLLNE	R_C13N15	1677.9071
	LacZ7-V8-L	RVNWLGLLPQE	R_C13N15	1324.7372
	LacZ8-V8-L	NGLRCLTRE	R_C13N15	1118.5735
	LacZ9-V8-L	GFHMLIGGD	F_C13N15	946.4451
	LacZ1-TrpV8-L	GVNSAFHLWCNLR	R_C13N15	1573.7692
	LacZ4-TrpV8-L	SALQLWLTVR	R_C13N15	1186.6943
	LacZ5-TrpV8-L	ALHISAWQQWR	R_C13N15	1395.7280
	LacZ6-TrpV8-L	AVLITTAHAWQHQLK	K_C13N15	1716.9544

[a] Amino acid residues at the Gly (GGG) codon are shown in red. Stable isotopically labeled amino acids are underlined.
