# Supplementary material for: Analysis of Genetic Code Ambiguity Arising from Nematode-Specific Misacylated tRNAs
Source: PLoS One. 2015 Jan 20;10(1):e0116981. doi: 10.1371/journal.pone.0116981 (PMC4300185; doi:10.1371/journal.pone.0116981)
Supplement: S4 Table — The peptide sequences containing amino acid residues (red) arising from the decoding of the GGG codon are listed. a Chromatographic retention times of the targeted peptides are also shown. (PDF) [file pone.0116981.s004.pdf]

Table S4. Summary of the identified peptides from transgenic worms expressing GFP–LacZ

Type	Sequence	Observed *m/z*	Mascot Score	Retention time (min) [a]		
				Candidate	Internal Standard	Δ time
GGG-to-Gly	VNWLGLGPQENYPDR	879.4347	46.83	61.3	61.1	0.2
	SAGQLWLTVR	565.8201	45.41	54.9	54.8	0.1
	AGHISAWQQWR	447.2277	21.26	43.7	43.7	0.0
	RVNWLGLGPQE	634.8417	49.94	58.9	58.8	0.1
GGG-to-Leu		Not identified				

The peptide sequences containing amino acid residues (red) arising from the decoding of the GGG codon are listed.

[a] Chromatographic retention times of the targeted peptides are also shown.
