# Supplementary material for: Analysis of Genetic Code Ambiguity Arising from Nematode-Specific Misacylated tRNAs
Source: PLoS One. 2015 Jan 20;10(1):e0116981. doi: 10.1371/journal.pone.0116981 (PMC4300185; doi:10.1371/journal.pone.0116981)
Supplement: S5 Table — (PDF) [file pone.0116981.s005.pdf]

Table S5. Oligonucleotides used to detect the CCA sequence at the 3′ end of each tRNA

Name	Type	Strand	Sequence
PA	anti-3’ adaptor	Reverse	GTCTCTAGCCTGCAGGATCGATG
P1	$tRNA^{Gly}$ (UCC)	Forward	GCATGGATGCCTTCCAAGC
P2	$tRNA^{Ile}$ (UAU)	Forward	GCGCGTGGTACTTATAATGC
P3	nev-$tRNA^{Gly}$ (CCC)	Forward	CCTATTCTGGTAACAGAGCG
P4	nev-$tRNA^{Ile}$ (UAU)	Forward	CTCATTGGGTTAAACCAGTCG
