# Supplementary material for: Analysis of Genetic Code Ambiguity Arising from Nematode-Specific Misacylated tRNAs
Source: PLoS One. 2015 Jan 20;10(1):e0116981. doi: 10.1371/journal.pone.0116981 (PMC4300185; doi:10.1371/journal.pone.0116981)
Supplement: S6 Table — a PCR reactions were performed using the indicated cycle numbers: denaturation for 10 s at 98°C, annealing for 3 s at the indicated temperatures and extension for 6 s at 74°C (See Materials and Methods for details). (PDF) [file pone.0116981.s006.pdf]

Table S6. Oligonucleotides used to analyze the subcellular localization of tRNAs

Name	Type	Strand	Sequence	Optimized PCR condition [a]	
				Annealing	Cycles
P5	snU6	Forward	GTTCTTCCGAGAACATATAC	55 °C	x 20
P6		Reverse	AACGCTTCACGAATTTGC		
P7	snoU3	Forward	ACTATACAGAATCATTTCTGCAG	60 °C	x 30
P8		Reverse	ACTGCTCAGAAGAGCAGG		
P9	$tRNA^{iMet}$	Forward	AGCAGCGTGGCGCAGTGGAA	60 °C	x 25
P10		Reverse	TAGCAGCGAGTGGTTTCGATCCA		
P11	$tRNA^{Gly}$ (UCC)	Forward	GCGTTCGTGGTGTAATGGTCAGC	60 °C	x 35
P12		Reverse	TGCGTTCGGGGGGAATCGAA		
P13	$tRNA^{Ile}$ (UAU)	Forward	GCCCCATTGGCGCAGTCGGTTAGC	60 °C	x 30
P14		Reverse	TGCCCCATGCCAGGCTCGAACTG		
P15	nev-$tRNA^{Gly}$ (CCC)	Forward	GCGGTGGTGGCCGAGCGGTCA	60 °C	x 35
P16		Reverse	CCCGCGCTCTGTTACCAGAATAG		
P17	nev-$tRNA^{Ile}$ (UAU)	Forward	GCCCCGGTGGCCGAGCGGTCG	60 °C	x 30
P18		Reverse	CCCGCGACTGGTTTAACCCAATGA		

[a] PCR reactions were performed using the indicated cycle numbers : denaturation for 10 s at 98 °C, annealing for 3 s at the indicated temperatures and extension for 6 s at 74 °C (See Materials and Methods for details).
