# Supplementary material for: Analysis of Genetic Code Ambiguity Arising from Nematode-Specific Misacylated tRNAs
Source: PLoS One. 2015 Jan 20;10(1):e0116981. doi: 10.1371/journal.pone.0116981 (PMC4300185; doi:10.1371/journal.pone.0116981)
Supplement: S1 Fig — MS/MS spectrum of the identified peptide from the GFP-LacZ proteins expressed in C. elegans was compared with those of synthesized peptides (IS) with the sequence SA(G/L)QLWLTVR. The C-terminal arginine residue of each IS was labeled with a stable isotope (Δm/z = 10.008269). (PDF) [file pone.0116981.s007.pdf]

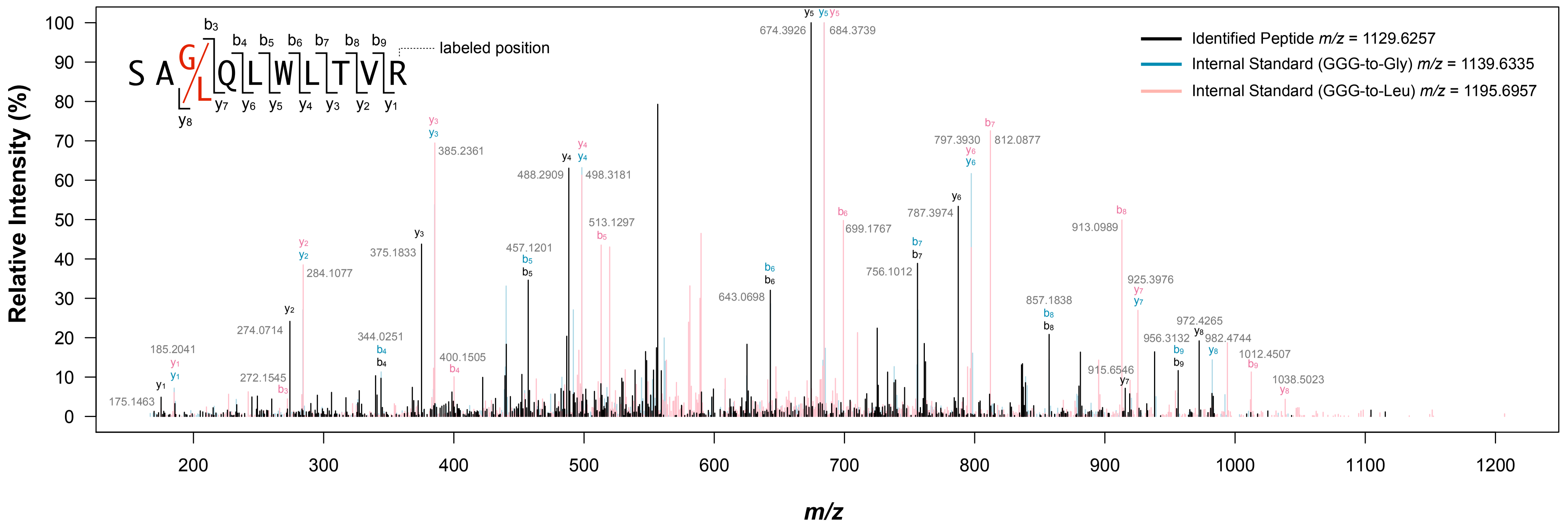


**Figure S1. Fragmentation pattern in the mass spectrum of the identified peptide from *C. elegans* is inconsistent with those of the peptides containing misincorporated Leu.** MS/MS spectrum of the identified peptide from the GFP-LacZ proteins expressed in *C. elegans* was compared with those of synthesized peptides (IS) with the sequence SA(G/L)QLWLTVR. The C-terminal arginine residue of each IS was labeled with a stable isotope ($\Delta m/z = 10.008269$).
